# Azacitidine or intensive chemotherapy for older patients with secondary or therapy-related acute myeloid leukemia

**DOI:** 10.18632/oncotarget.15988

**Published:** 2017-03-07

**Authors:** Pierre-Yves Dumas, Sarah Bertoli, Emilie Bérard, Clémence Médiavilla, Edwige Yon, Suzanne Tavitian, Thibaut Leguay, Françoise Huguet, Edouard Forcade, Noël Milpied, Audrey Sarry, Mathieu Sauvezie, Pierre Bories, Arnaud Pigneux, Christian Récher

**Affiliations:** ^1^ Service d’Hématologie Clinique et de Thérapie Cellulaire, Centre Hospitalier Universitaire de Bordeaux, Hôpital Haut-Lévèque, Bordeaux, France; ^2^ Université de Bordeaux, France; ^3^ U1035 INSERM, Bordeaux, France; ^4^ Service d’Hématologie, Centre Hospitalier Universitaire de Toulouse, Institut Universitaire du Cancer de Toulouse Oncopole, Toulouse, France; ^5^ Université Toulouse III Paul Sabatier, Toulouse, France; ^6^ Cancer Research Center of Toulouse, UMR1037-INSERM, ERL5294-CNRS, Toulouse, France; ^7^ Service d’Epidémiologie, Centre Hospitalier Universitaire de Toulouse, Toulouse, France; ^8^ UMR 1027, INSERM-Université de Toulouse III, Toulouse, France; ^9^ Oncomip Network, Réseau de Cancérologie de Midi-Pyrénées, Toulouse, France

**Keywords:** therapy-related acute myeloid leukemia, secondary AML, azacitidine, intensive chemotherapy, older patients

## Abstract

The treatment of older patients with acute myeloid leukemia that is secondary to previous myelodysplastic syndrome, myeloproliferative neoplasm, or prior cytotoxic exposure remains unsatisfactory. We compared 92 and 107 patients treated, respectively, with intensive chemotherapy or azacitidine within two centres. Diagnoses were 37.5% post-myelodysplastic syndrome, 17.4% post-myeloproliferative neoplasia, and 45.1% therapy-related acute myeloid leukemia. Patients treated by chemotherapy had less adverse cytogenetics, higher white blood-cell counts, and were younger: the latter two being independent factors entered into the multivariate analyses. Median overall-survival times with chemotherapy and azacitidine were 9.6 (IQR: 3.6−22.8) and 10.8 months (IQR: 4.8−26.4), respectively (*p* = 0.899). Adjusted time-dependent analyses showed that, before 1.6 years post-treatment, there were no differences in survival times between chemotherapy and azacitidine treatments whereas, after this time-point, patients that received chemotherapy had a lower risk of death compared to those that received azacitidine (adjusted HR 0.61, 95%CI: 0.38−0.99 at 1.6 years). There were no interactions between treatment arms and secondary acute myeloid leukemia subtypes in all multivariate analyses, indicating that the treatments had similar effects in all three subtypes. Although a comparison between chemotherapy and azacitidine remains challenging, azacitidine represents a valuable alternative to chemotherapy in older patients that have secondary acute myeloid leukemia because it provides similar midterm outcomes with less toxicity.

## INTRODUCTION

The subgroup of patients with non *de novo* acute myeloid leukemia (AML) is frequently and improperly named as having “secondary” AML. This group is heterogeneous and encompasses both therapy-related acute myeloid leukemia (t-AML), which occurs after prior exposure to cytotoxic chemotherapy and/or radiotherapy, and secondary AML (sAML), which occurs in the course of a previous myeloid disease such as myelodysplastic syndrome (MDS) or Philadelphia-negative myeloproliferative neoplasm (MPN) [1].

In most studies, both t-AML and sAML have been associated with a worse prognosis compared to *de novo* AML, although subgroups of patients with a better outcome have been reported [2-4]. Many well-known adverse factors are more frequently observed in patients with t-AML and sAML, which can explain the worse outcome: i.e., older age, comorbidities, multilineage dysplasia, and poor-risk cytogenetics [3]. Because both t-AML and sAML are often excluded from prospective trials, an optimal treatment remains to be established. In patients deemed fit for intensive therapy, which represents less than half of this older-patient population, therapeutic strategies differ little from those for *de novo* AML: these patients are offered induction chemotherapy and allogeneic stem-cell transplantation (HSCT).

Recent data from a Danish registry showed that MDS-sAML and tAML are independently associated with increased risk of death, although this effect was less pronounced in patients > 60 years and in patients with adverse or intermediate cytogenetics. However, AML secondary to MPN or chronic myelomonocytic leukemia (CMML) were associated with worse overall survival independent of cytogenetics and age [5]. The overall prognosis remains very poor, with median overall survival (OS) of less than six months, indicating that alternative treatments are needed [6].

Azacitidine has been recently approved in Europe for older AML patients and now offers a reasonable alternative to intensive chemotherapy, at least for a subset of patients [7]. We have previously shown in a series of 95 older AML patients, that most benefit was observed in patients that had a low white blood-cell count (WBC), a good performance status, and intermediate-risk cytogenetics [8]. In addition, when compared with conventional care regimen, azacitidine appeared better in patients of the adverse cytogenetic risk group [7]. It is noteworthy that in most studies that have focused on azacitidine treatment for AML, neither sAML nor t-AML has emerged as having worse risk factors [5-11]. Moreover, azacitidine has shown efficacy in subgroups that have features frequently encountered in t-AML or sAML, including multilineage dysplasia and adverse-risk cytogenetics [7, 8]. In this study, we compared the outcomes of patients aged > 60 years with t-AML or sAML and that had received intensive chemotherapy or azacitidine.

## RESULTS

### Patients and treatments

This study included 199 patients selected to receive intensive chemotherapy (*n* = 92) or azacitidine (*n* = 107). The median year of treatment was 2011 in the intensive arm and 2010 in the azacitidine arm. The patients’ characteristics are depicted in Table 1. There were 69 cases of post-MDS AML (37.5%), 32 cases of post-MPN AML (17.4%), and 83 cases of t-AML (45.1%). There were no clinical differences between the chemotherapy and azacitidine groups in terms of performance status and the Charlson comorbidity index. In the azacitidine group, patients were older, had lower WBC counts, and more frequently had post-MDS AML and adverse cytogenetics, including a monosomal karyotype, compared to the chemotherapy group.

**Table 1:** Patients characteristics

	All patients N=199 (100%)	Azacitidine N=107 (53.8%)	Intensive chemotherapy N=92 (46.2%)	p
**Age (years)** Median (IQR) Range	72.0 (65.9-77.5) 60.6, 87.8	76.5 (71.4-80.6) 60.9, 87.8	66.6 (63.5-71.4) 60.6, 83.1	p<0.0001
**Age (years)** <70 ≥70	85 (42.7) 114 (57.3)	20 (18.7) 87 (81.3)	65 (70.7) 27 (29.3)	p<0.0001
**Male gender-n (%)**	117 (58.8)	62 (57.9)	55 (59.8)	p=0.80
**sAML subtype-n (%)** Post-MDS Post-MPN Therapy- related	69 (37.5) 32 (17.4) 83 (45.1)	52 (50.5) 18 (17.5) 33 (32.0)	17 (21.0) 14 (17.3) 50 (61.7)	p<0.0001
**Performance status-n (%)** 0-1 2-3	123 (71.5) 49 (28.5)	59 (67.8) 28 (32.2)	64 (75.3) 21 (24.7)	p=0.28
**Charlson comorbidity index-n (%)** 0 ≥1	59 (33.7) 116 (66.3)	32 (36.8) 55 (63.2)	27 (30.7) 61 (69.3)	p=0.40
**Infection at diagnosis-n (%)** Yes No	27 (15.1) 152 (84.9)	18 (20.5) 70 (79.5)	9 (9.9) 82 (90.1)	p=0.048
**WBC (G/L)** N Median [IQR] Range	197 4.2 [ 1.8-18.3] 0.5,433.0	105 2.4 [ 1.4- 5.8] 0.6,91.4	92 14.1 [ 3.6-62.7] 0.5,433.0	p<0.0001
**WBC ≥ 15 G/L-n (%)** Yes No	54 (27.4) 143 (72.6)	9 (8.6) 96 (91.4)	45 (48.9) 47 (51.1)	p<0.0001
**Bone marrow blasts (%)** Median [IQR] Range	35.0 [25.0-62.0] 5.0,98.0	30.0 [21.0-46.0] 9.0,79.0	50.0 [27.5-82.0] 5.0,98.0	p<0.0001
**Cytogenetics - n (%)** Favorable Intermediate Unfavorable /non monosomal Monosomal karyotype	0 126 (64.0) 46 (23.4) 25 (12.6)	0 61 (58.1) 24 (22.9) 20 (19.0)	0 65 (70.7) 22 (23.9) 5 (5.4)	p=0.005
**Serum ferritin (µgrams/L)** N Median [IQR] Range	108 747 [411-1384] 41,38950	47 537 [285-1204] 41,4015	61 1045 [494-1598] 49,38950	p=0.023
**Serum albumin (grams/L)** N Median [IQR] Range	150 39.0 [35.4-42.0] 19.0,57.3	72 40.0 [36.9-43.0] 25.0,46.0	78 37.2 [34.4-41.0] 19.0,57.3	p=0.036
**LDH (UI/L)** N Median [IQR] Range	168 574.5 [369-998] 136,12806	80 481 [346-641] 170,3525	88 738.5 [419.5-1509] 136,12806	p=0.002

Abbreviations: IQR: interquartile range, sAML: secondary AML; MDS: Myelodysplastic syndrome, MPN: Myeloproliferative neoplasm, WBC: white blood cell count.

The median times from diagnosis to initiation of treatment were 0.7 month (interquartile range (IQR): 0.4-1.5) for azacitidine and 0.2 (IQR: 0.1-0.5) for chemotherapy (*p* < 0.0001). In the azacitidine group, patients received a median number of seven cycles (IQR, 2-15), using the standard 7-day scheme in 83% of cases. In the chemotherapy group, 60% of patients received a three-drug schedule that combined idarubicin, standard-dose cytarabine, and lomustine (Supplementary Table 1). Among the 92 patients who received intensive chemotherapy, 47 achieved complete remission (CR): 43 after a single induction course and four after a second induction regimen with high-dose cytarabine. Twenty patients were refractory to intensive chemotherapy and were then managed by best supportive care without receiving hypomethylating agents. One patient underwent HSCT in a refractory situation. Thirty-four CR patients were treated during the post-remission phase: 14 with at least one course of intermediate-dose cytarabine (≥1 g/m^2^) followed by HSCT given to 5 patients, 18 patients received a low-intensity regimen of chemotherapy, and 2 patients received HSCT without any consolidation.

Among the 107 patients who received azacitidine, only one underwent HSCT. This patient is still alive at 49 months (Table 2). The median time between response to chemotherapy and the end of post-remission-treatment was 4.4 months (IQR: 2.1-8.4). None of the patient that received azacitidine as first treatment in our cohort received intensive chemotherapy as a second-line treatment, whereas only three patients in the chemotherapy group received a hypomethylating agent at relapse.

**Table 2:** Response and outcome according to treatment arm

	Azacitidine N=107	Intensive chemotherapy N=92	p
Overall response (CR+CRi)-n (%)	21 (19.6%)	58 (63.0%)	<0.0001
CR-n (%)	12 (11.2%)	47 (51.1%)	<0.0001
CRi-n (%)	9 (8.4%)	11 (12.0%)	0.4070
PR-n (%)	7 (6.5%)	NA	
HI-n (%) -Overall -1 lineage -2 lineages -3 lineages	21 (19.6%) 10 (9.4%) 8 (7.5%) 3 (2.8%)	NA	
Day-30 deaths-n (%) Day-60 deaths-n (%)	6 (5.6%) 19 (18.3%)	10 (10.9%) 16 (17.4%)	0.1930 0.8727
Causes of early deaths ^a^ -n (%) -AML progression -Infection -Cardiac event -Hemorrhage -Unknown	6 (5.6%) 1 (0.9%) 0 0 0	5 (5.4%) 4 (4.3%) 0 0 2 (2.2%)	
AlloSCT-n (%)	1 (1.1%)	8 (8.7%)	0.0348

Abbreviations: CR: complete response, CRi: complete response with incomplete blood recovery, PR: partial response, HI: Hematological improvement. a. Early death within 30 days from treatment (several causes are possible by patient), NA: Not applicable, AlloSCT: allogeneic stem-cell transplantation.

### Assessment of choice criteria between azacitidine and chemotherapy

Univariate analyses showed that the main factors significantly associated with the choice of whether patients received chemotherapy or azacitidine were age, WBC count, and cytogenetic risk, especially if there was a monosomal karyotype (Table 3). In the multivariate analyses, the two mains factors regarding choice of treatment were age and WBC count, meaning that the probability of receiving chemotherapy was significantly decreased with age (adjusted OR 0.26, 95%CI: 0.18-0.39 for each increase of 5 years of age, *p* < 0.001), and an increased WBC count (adjusted OR 17.54, 95%CI: 6.13-50.2 for patients with a WBC count ≥15 G/L vs. < 15 G/L; *p* < 0.001) (Table 3).

**Table 3:** Univariate and multivariate analyses of factors associated with the choice of treatment

	Univariate analysis					Multivariate analysis		
	N	N Events	OR	95% CI	p*	aOR	95% CI	p*
**Age as continuous variable** Interval: 5 years	199	92	0.31	[0.22;0.43]	<0.001	0.26	[0.18;0.39]	<0.001
**Age (years)** <70 ≥70	85 114	65 27	1 0.10	- [0.05;0.19]	- <0.001			
**Performance status** 0-1 2-3	123 49	64 21	1 0.69	- [0.35;1.35]	- 0.278			
**Charlson comorbidity index** 0 >0	59 116	27 61	1 1.31	- [0.70;2.46]	- 0.394			
**WBC (G/L)** <15 ≥15	143 54	47 45	1 10.21	- [4.61;22.64]	<0.001	1 17.54	- [6.13;50.21]	<0.001
**Cytogenetics** Intermediate Unfavorable /non monosomal Monosomal karyotype	126 46 25	65 22 5	1 0.86 0.23	- [0.44;1.69] [0.08;0.66]	- 0.662 0.006			

Abbreviations: WBC: white blood cell count, OR: Odds ratio, CI: confidence interval, *p value for factors assessed in the decision criteria for chemotherapy (versus azacitidine).

### Safety

There were 10 (10.9%) early deaths in patients treated by chemotherapy and 6 (5.8%) in patients that received azacitidine (*p* = 0.193) (Table 2). Univariate analyses of the factors that influenced early death are shown in Supplementary Table 2. In the multivariate analyses, only AML subtype and type of treatment remained significantly predictive of early death, meaning that t-AML was associated with a lower risk of death (vs. post-MDS AML, adjusted OR 0.08, 95%CI: 0.02-0.43; *p* = 0.003) and chemotherapy was associated with a higher risk (vs. azacitidine, adjusted OR 3.80, 95%CI: 1.17-12.4; *p* = 0.026) (Table 4).

**Table 4:** Multivariate analyses for early death, response and overall survival

	N	N Events	aOR/aHR	95% CI	p
	*Early deaths*				
**Subtype of sAML** MDS-related MPN-related Therapy-related	67 32 82	10 2 2	1 0.28 0.08	- [0.05;1.43] [0.02;0.43]	- 0.126 0.003
**Treatment arm** Azacitidine Intensive chemotherapy	104 92	6 10	1 3.80	- [1.17;12.36]	- 0.026
	***Response to treatment***				
**Age as continuous variable** Interval of 5 years	196	78	0.67	[0.50;0.90]	0.008
**Treatment arm** Azacitidine Intensive chemotherapy	104 92	20 58	1 4.09	- [1.94;8.63]	- <0.001
	***Overall survival***				
**Ferritinemia (µg/L)** <400 400-750 750-1400 ≥1400	25 29 26 27	15 23 20 25	1 1.57 1.46 3.70	- [0.80;3.05] [0.73;2.94] [1.89;7.22]	- 0.188 0.287 <0.001
**LDH (U/L)** Normal Elevated ^a^	48 120	37 100	1 1.87	- [1.25;2.82]	- 0.003
**Cytogenetics** Intermediate Unfavorable /non monosomal Monosomal karyotype	126 46 25	98 43 22	1 2.50 4.20	- [1.71;3.67] [2.51;7.02]	- <0.001 <0.001
**Treatment arm** **Azacitidine** **Intensive chemotherapy**	105 92	92 71	See Figure 1C		

Abbreviations: sAML: secondary AML, MDS: Myelodysplastic syndrome, MPN: Myeloproliferative neoplasm, aOR: adjusted odds ratio, aHR: adjusted hazard ratio, CI: confidence interval, a. rate above the benchmark.

### Efficacy

Overall response (CR+CRi) was documented in 58 patients (63%) that received chemotherapy and in 21 patients (19.6%) that received azacitidine (*p* < 0.0001) (Table 2). In the azacitidine group, seven additional patients (6.5%) achieved a partial response (PR) and 21 patients (19.6%) that were classified as failure with the IWG-AML criteria achieved a major hematological improvement. The median delay before the best response to azacitidine was 5.7 months (IQR: 5.2-8.9). Univariate analyses on the factors that influenced response to treatment are shown in Supplementary Table 3.

In the multivariate analyses, only age and type of treatment remained significantly associated with response, meaning that the probability of response decreased with age (adjusted OR 0.67, 95%CI: 0.50-0.90 for each increase in 5 years of age, *p* = 0.008) and increased in patients treated with chemotherapy (adjusted OR 4.09, 95%CI: 1.94-8.63; *p* < 0.001) (Table 4).

### Overall survival

With a median follow-up period of 3.4 years (IQR: 2.1-5.4), the median OS time for the entire cohort was 10.8 months (IQR: 4.8-26.4). Median OS with chemotherapy was 9.6 months (IQR: 3.6-22.8) and with azacitidine it was 10.8 months (IQR: 4.8-26.4, *p* = 0.899). Univariate analyses of the factors associated with OS are shown in Supplementary Table 4.

**Figure 1:**
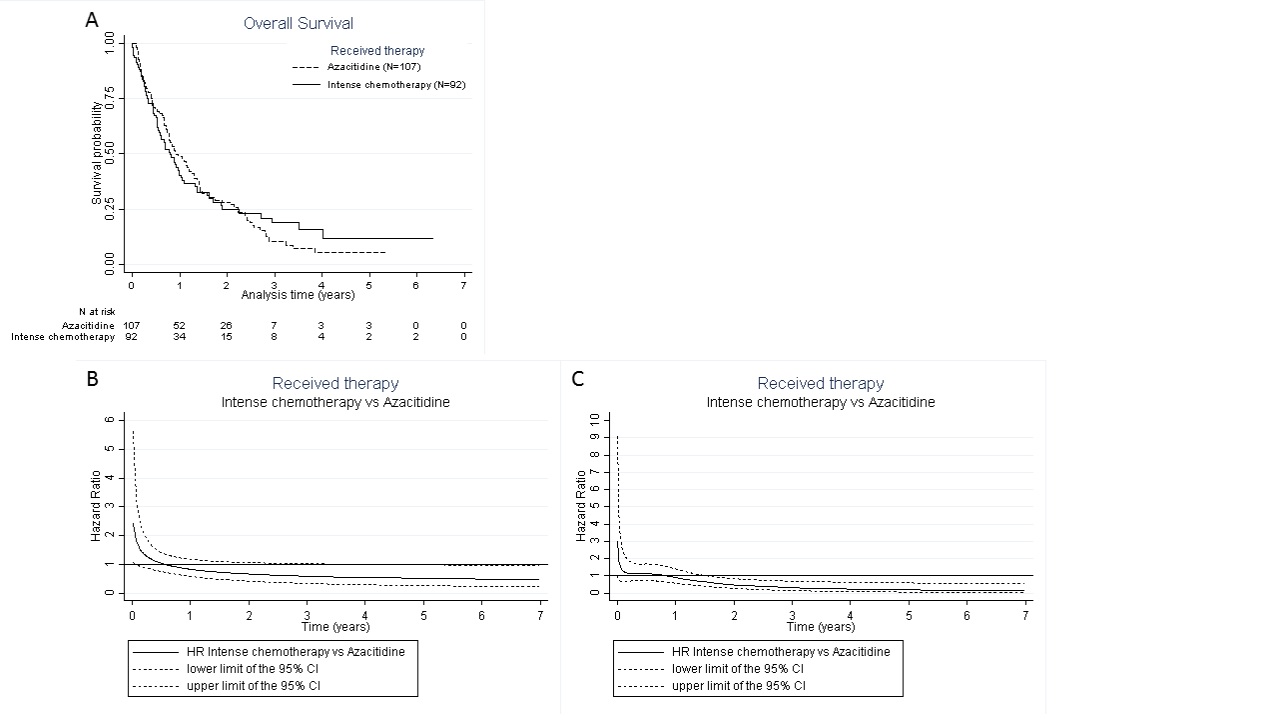
A Kaplan-Meier curve for overall survival according to treatment. **B.** Royston and Parmar non-adjusted hazard ratio for overall survival after treatment with azacitidine vs. chemotherapy for each year after diagnosis. Before 15 days of follow-up, patients treated with intensive chemotherapy had a significantly higher risk of death compared to those that received azacitidine. At day 15, the risk of death was higher in the intensive-chemotherapy group compared to the azacitidine group (HR 2.22, 95%CI: 1.03-4.75). Beyond 15 days of follow-up, there was no significant difference in survival between the two groups. **C.** Royston and Parmar adjusted hazard ratio for overall survival after treatment with azacitidine vs. chemotherapy for each year after diagnosis. Before 1.6 years of follow-up, there was no significant difference in survival between the two groups. After 1.6 years, patients treated with intensive chemotherapy had a significantly reduced risk of death compared to those that received azacitidine (aHR 0.61, 95%CI: 0.38-0.99). Interaction between azacitidine vs. chemotherapy and the AML subtypes (t-AML, post-MDS, or post-MPN AML) was not significant, showing that the effect of azacitidine vs. chemotherapy was not significantly different according to AML subtypes. So, there is no indication to stratify the analysis on AML subtypes (Figure C was the same for t-AML, post-MDS, or post-MPN AML).

The Kaplan-Meier curve showed a time-dependent effect according to treatment arm, indicating that the log-rank test could not adequately compare the treatments (Figure 1A). Thus, we used the Royston and Parmar model, which took into account the interactions between time and treatment effect, and allowed graphical representation of the unadjusted risk of death, as shown in Figure 1B. Multivariate analysis of the factors associated with survival according to this model showed that serum ferritin ≥1400 µg/L (adjusted OR 3.70, 95%CI: 1.89-7.22 vs. < 400 µg/L; *p* < 0.001), elevated lactate dehydrogenase (adjusted OR 1.87, 95%CI: 1.25-2.82; *p* = 0.003), and cytogenetic risk (adjusted OR 2.50, 95%CI: 1.71-3.67; *p* < 0.001 for adverse non-monosomal, and adjusted OR 4.20, 95%CI: 2.51-7.02; *p* < 0.001 for monosomal karyotype, both vs. intermediate karyotype) were significantly associated with worse OS (Table 4). Changes in the HR for death with chemotherapy vs. azacitidine, and adjusted for the covariables in the multivariate model, are shown in Figure 1C: this indicates that before 1.6 years, there were no difference in survival between chemotherapy and azacitidine treatments whereas, after this time-point, patients that received chemotherapy had a lower risk of death compared to those that received azacitidine (adjusted HR 0.61, 95%CI: 0.38-0.99 at 1.6 years).

### Characteristics and outcomes of patients with t-AML, post-MDS, or post-MPN AML

The characteristics of patients according to AML subtype are shown in Table 5. Patients with post MDS-AML were older and more often received azacitidine, whereas patients with post-MPN had a higher-risk cytogenetics, including the monosomal karyotypes. There was no interaction between the treatment arms and AML subtypes in all the multivariate analyses, indicating that the effects of treatment were similar for all three groups of AML.

**Table 5:** Characteristics of patients according to AML subtypes

	Post-MDS AML N=69 (37.5%)	Post-MPN AML N=32 (17.4%)	tAML N=83 (45.1%)	P
**Age (years)** Median (IQR) Range	75.2 (68.4-79.9) 61.0,86.6	70.5 (65.0-75.3) 61.2,83.8	70.1 (64.5-74.9) 60.6,87.8	0.0018
**Age (years)** <70 ≥70	21 (30.4) 48 (69.6)	16 (50.0) 16 (50.0)	40 (48.2) 43 (51.8)	0.0510
**Male gender-n (%)**	47 (68.1)	17 (53.1)	42 (50.6)	0.0800
**Performance status-n (%)** 0-1 2-3	42 (76.4) 13 (23.6)	15 (53.6) 13 (46.4)	59 (75.6) 19 (24.4)	0.0560
**Charlson comorbidity index-n (%)** 0 >0	23 (37.1) 39 (62.9)	14 (53.9) 12 (46.2)	17 (22.1) 60 (77.9)	0.0080
**Infection at diagnosis-n (%)** Yes No	9 (14.7) 52 (85.3)	5 (19.2) 21 (80.8)	12 (15.0) 68 (85.0)	0.8790
**WBC (G/L)** N Median (IQR) Range	68 3.1 (1.7-8.8) 0.6,168.4	32 5.0 (3.0-13.0) 0.9,80.1	83 3.8 (1.5-27.2) 0.5,122.8	0.3071
**WBC ≥ 15 G/L-n (%)** Yes No	12 (17.7) 56 (82.4)	6 (18.8) 26 (81.3)	25 (30.1) 58 (69.9)	0.1560
**Bone marrow blasts (%)** Median (IQR) Range	29 (22-45) 20,95	32 (21-40) 12,85	52 (29-76) 5,98	0.0001
**Cytogenetics - n (%)** Favorable Intermediate Unfavorable /non monosomal Monosomal karyotype	0 (0.0) 50 (74.6) 13 (19.4) 4 (6.0)	0 (0.0) 13 (40.6) 11 (34.4) 8 (25.0)	0 (0.0) 51 (61.5) 20 (24.1) 12 (14.5)	0.0140
**Serum ferritin (µgrams/L)** N Median (IQR) Range	42 547 (336-1310) 72,38950	14 1103 (597-1538) 41,9750	42 768 (438-1304) 49,3252	0.3397
**Serum albumin (grams/L)** N Median (IQR) Range	54 40 (36-42) 19,46	20 37 (34-41) 22,48	65 38 (35-42) 21,57	0.2551
**LDH (UI/L)** N Median (IQR) Range	59 572 (402-792) 170,12806	23 836 (448-1735) 229,3525	75 494 (332-774) 136,7258	0.0512
**Treatment** Intensive Chemotherapy Azacitidine	17 (24.6) 52 (75.4)	14 (43.8) 18 (56.2)	50 (60.2) 33 (39.8)	<0.0001
**Response – n (%)** All Chemotherapy Azacitidine	19 (27.5) 9 (52.9) 10 (19.2)	10 (31.3) 7 (50.0) 3 (16.7)	45 (54.2) 38 (76.0) 7 (21.2)	0.0019 0.0790 1.0000
**Early death -n (%)** All Chemotherapy Azacitidine	10 (14.9) 4 (23.5) 6 (12.0)	2 (6.3) 2 (14.3) 0 (0.0)	2 (2.4) 2 (4.0) 0 (0.0)	0.0126 0.0350 0.0430
**Median OS-months (IQR)** All Chemotherapy Azacitidine	13.2 (3.6-33.6) 7.2 (2.4-26.4) 13.2 (3.6-33.6)	8.4 (4.8-16.8) 7.2 (3.6-19.2) 8.4 (4.8-13.2)	10.8 (6.0-22.8) 10.8 (6.0-22.8) 13.2 (7.2-25.2)	0.1230 0.8425 0.0690

Abbreviations: MDS: Myelodysplastic syndrome, MPN: Myeloproliferative neoplasm, tAML: therapy related AML, IQR: interquartile range, WBC: white blood cell count.

## DISCUSSION

In this study, we have described one of the largest cohorts of t-AML and sAML patients aged ≥60 years and selected from daily practice for two different treatment approaches over a 7-year period. We show that patients that received intensive chemotherapy had a better complete response rate, a higher early death rate, and similar median OS times compared to patients that received azacitidine. However, as the risk of death varied over time according to treatment, we compared the survival of patients treated by azacitidine versus intensive chemotherapy using time-dependent analyses and showed that, after adjustment for the main prognostic factors, patients that received intensive chemotherapy had better survival times after 1.6 years post-diagnosis.

The AZA-AML-001 randomized trial compared azacitidine versus conventional-care regimen in AML patients aged ≥65 years and with a WBC count of < 15 G/L. The investigators chose between intensive chemotherapy, low-dose cytarabine, and best supportive care [7]. This international multicenter trial included 488 patients, of whom only 88 were selected for randomization versus chemotherapy, indicating that only a minority of physicians considered this issue of relevance. This suggests that patients are primarily selected to receive intensive chemotherapy and only thereafter for other strategies if patients are deemed unfit for chemotherapy because of the comorbidities, adverse cytogenetics, or the patient’s choice. Based on the subjective criteria used to select treatment in routine practice, we have previously identified three distinct groups of AML patients aged > 60 years [8]: i.e., (i) the intensive-chemotherapy group included the “youngest” patients with proliferative, *de novo* AML, or non-adverse cytogenetics, (ii) the group that received a hypomethylating agent included patients with a low WBC count, secondary AML, and adverse cytogenetics, and (iii) the best supportive-care group included the “oldest” patients that also had proliferative AML. In the present study, it seems that this same selection was applied to patients with t-AML and sAML, at least regarding the choice between chemotherapy and azacitidine, as patients selected for chemotherapy had less adverse cytogenetics, higher WBC counts, and were younger, with the latter two factors being the independent factors of choice in our multivariate analyses.

Reflecting the difficulty in including sufficient numbers of cases to make a thorough comparison between chemotherapy and azacitidine, we could only match 18 patients from each group based on the propensity score method, which precluded any relevant analysis in these matched subgroups. However, our adjusted analyses were robust enough to support our findings. Indeed, although we cannot avoid the biases inherent in this observational (non-randomized) study, we used multivariate analyses adjusted for all parameters known to influence the outcomes in AML. Finally, we can at least infer that the midterm prognosis of older patients with higher risk disease treated by azacitidine is similar to that of younger patients selected for intensive chemotherapy.

Overall, azacitidine appeared to be a reasonable alternative to chemotherapy as it provided similar midterm outcomes with less toxicity when compared to chemotherapy in patients aged ≥60 years that had t-AML/sAML, similar *de novo* AML [5, 7, 8]. Finally, we acknowledge that the impact of both therapeutic strategies on the quality of life (QOL) is a key point. Although we cannot provide data on QOL because of our study’s design, no clear difference in QOL was found between azacitidine and conventional care in the AZA-AML-001 trial.

In our series, ∼80% of patients had a high level of serum ferritin, and hyperferritinemia was independently associated with lower OS. As a marker of red blood cell transfusion burden, increased serum ferritin levels have been associated with worse outcomes in MDS and sAML because of the negative impact of iron overload [12,13]. We have previously shown that hyperferritemia also impacts on the OS of younger patients with *de novo* AML who do not have post-transfusion iron-overload at diagnosis [14]. Serum ferritin could thus affect the prognosis of *de novo* and secondary AML through multiple mechanisms, including resistance to chemotherapy and likely also azacitidine.

In conclusion, the prognosis of secondary AML remains very poor, with future therapies and progress urgently needed. New therapeutic strategies that include hypomethylating agents or chemotherapy combined with novel drugs are being intensively trialed [15,16]. Such therapies should hopefully improve the outcomes for this difficult-to-treat AML population [17].

## MATERIALS AND METHODS

### Patients and treatments

The selection criteria for this retrospective study were the following: diagnosis of AML according to WHO criteria [1] (excluding acute promyelocytic leukemia and core-binding factor AML) that were made between January 1^st^, 2007 and December 31^st^ 2013 in Toulouse or Bordeaux University Hospitals. Patients were aged ≥60 years and had no previous treatment, except for hydroxyurea, secondary to (i) MDS/CMML diagnosed more than 3 months before AML (post MDS-AML), (ii) Philadelphia-negative MPN (post-MPN AML), or (iii) prior exposure to chemotherapy or radiotherapy (t-AML). All patients with post MDS-AML that had previously received azacitidine for MDS were excluded from the study.

The center’s policies slightly differed: in Toulouse center, an adaptive approach was applied in routine practice based on initial characteristics such as white blood cell count, cytogenetics, age, secondary AML, performance status and comorbidities. Briefly, the first issue was to judge if patients could benefit from intensive chemotherapy (*i.e* < 75years, favorable/intermediate cytogenetic risk, *de novo* AML). If not, the second issue was to determine if patients could benefit from azacitidine (regardless of the BM blast percentage) with a special attention paid to proliferative AML since high WBC was already described as a poor prognostic factor in patients treated by azacitidine, as previously described [8]. In Bordeaux center, same evaluation based on initial characteristics such as white blood cell count, cytogenetics, age, secondary AML, performance status and comorbidities was also applied but azacitidine was performed only for patients unfit with < 30% BM blasts.

Written informed consent was obtained in accordance with the Declaration of Helsinki, to allow the collection of clinical data from an anonymized database, registered at the Commission Nationale de l’Informatique et des Libertés (CNIL) under access No. 1778920. These data were retrospectively collected until November 2011 from Toulouse and until November 2012 from Bordeaux, and were then prospectively collected thereafter. Classification of cytogenetic risk was defined according to the MRC classification [18]. Data on comorbidities were collected according to the Charlson comorbidity index [19]. The regimens of intensive induction chemotherapy and azacitidine are detailed in Supplementary Table 1.

### Assessment of safety and efficacy

See Supplementary File.

### Statistical analyses

Before doing any analysis, we assessed the power of the study: 165 deaths (94 treated by azacitidine and 71 by chemotherapy) provided a power of > 80% to detect a hazard ratio (HR) of death of ≥1.6 (for azacitidine vs. chemotherapy), with a two-sided type-1 error rate of 5% (α = 0.05), for the comparison of two exponential survival distributions [20]. Statistical analyses were performed using STATA statistical software, release 11.2 (STATA Corp., College Station, TX). We described the patients’ characteristics using numbers and frequencies for qualitative data, and medians, inter-quartile ranges (IQR), and ranges (minimum−maximum) for quantitative data. Comparisons between the patients’ characteristics were assessed using Student’s t-test or ANOVA (Mann-Whitney or Kruskall-Wallis test when the distribution departed from normality or when homoscedasticity was rejected) for continuous variables, and the χ^2^-test (or Fisher’s exact test when there were small expected numbers) for categorical variables. Assessment of independent-choice criteria between azacitidine and chemotherapy was based on a logistic regression model. Comparison of OS after azacitidine vs*.* chemotherapy was assessed using Kaplan-Meier curves and the log-rank test in the univariate analyses. Because the proportional-hazards assumption was not respected for treatments (azacitidine vs. chemotherapy), we used a Royston and Parmar survival model [21]. Differences in early death and response rate were compared between treatments using a logistic regression model. Included in the multivariate analyses were variables (particularly differences between azacitidine and chemotherapy groups) that had a *p*-value of < 0.20 in the univariate analyses and remained significantly and independently associated with OS, early death, or response rate (*p*-value < 0.05), after backward analysis. Allogeneic stem-cell transplantation was evaluated as a time-dependent potential confounder. Interactions between azacitidine vs. chemotherapy and the independent covariates (particularly the AML subtypes, t-AML, post-MDS, or post-MPN AML) were tested in the final models. None were significant. All reported *p*-values were two-sided and the significance threshold was < 0.05.

### Authorship

PYD, SB, CM, ST, TL, FH, EF, AS, MS, PB, and CR performed the research; PYD, SB, EB, NM, AP, and CR designed the research study; PYD, SB, EY, and EB analyzed the data; PYD, SB, EB, and CR wrote the paper.

## SUPPLEMENTARY MATERIALS TABLES


