# Supplementary material for: Azacitidine or intensive chemotherapy for older patients with secondary or therapy-related acute myeloid leukemia
Source: Oncotarget. 2017 Mar 7;8(45):79126–36. doi: 10.18632/oncotarget.15988 (PMC5668025; doi:10.18632/oncotarget.15988)
Supplement: Supplementary file 1 [file oncotarget-08-79126-s001.pdf]

Azacitidine or intensive chemotherapy for older patients with secondary or therapy-related acute myeloid leukemia

Supplementary Materials

Assessment of safety and efficacy

Early death (ED) was defined as death that occurred during the first 30 days after diagnosis. Response was assessed according to the International Working Group (IWG) on acute myeloid leukemia (AML).[1] Bone marrow assessment in patients treated with intensive chemotherapy was performed after blood recovery or if recovery was delayed until days 35 to 45. In the azacitidine group, bone marrow aspiration was carried out after 3 to 6 cycles. Patients that harbored a stable or a progressive disease were classified as failures according to IWG-AML criteria. Patients with a stable disease, hematological improvement (HI) either on erythroid, neutrophil or platelet lineage, was assessed according to the 2006 IWG–MDS criteria.[2] Overall survival (OS) was defined as the time from diagnosis until death from any cause or the date of last contact.

1. Cheson BD, Bennett JM, Kopecky KJ, et al. Revised recommendations of the International Working Group for Diagnosis, Standardization of Response Criteria, Treatment Outcomes, and Reporting Standards for Therapeutic Trials in Acute Myeloid Leukemia. J Clin Oncol 15 Dec 2003;21(24):4642-469.

2. Cheson BD, Greenberg PL, Bennett JM, et al. Clinical application and proposal for modification of the International Working Group (IWG) response criteria in myelodysplasia. Blood 15 July 2006;108(2):419-425.

Supplementary Table 1: Schema of induction chemotherapy and azacitidine

**Induction chemotherapy**
Cytarabine 200 mg/m^2/d for 7 days + Daunorubicin 60 mg/m^2/d for 3 days
Cytarabine 100 mg/m^2/d for 7 days + Idarubicin 8 mg/m^2/d for 5 days
Cytarabine 100 mg/m^2/d for 7 days + Idarubicin 8 mg/m^2/d for 5 days + L.200 mg/m^2 at day 1
**Azacitidine**
75 mg/m^2/d for 7 days on a 5 days on/2 days off/2 days on-basis every 28 days
75 mg/m^2/d for 5 days every 28 days for the frailest patients
until disease progression, unacceptable toxicity, or patient decision to withdraw

Supplementary Table 2: Univariate analysis of factors associated with early death

	N	N Events	OR	95% CI	p
**Age (years)**					
<70	84	7	1	-	-
≥70	112	9	0.96	[0.34;2.69]	0.940
**Sex**					
Male	115	11	1	-	-
Female	81	5	0.62	[0.21;1.86]	0.397
**Performance status**					
0-1	121	8	1	-	-
2-3	49	2	0.6	[0.12;2.94]	0.529
**Ferritinemia (µg/L)**					
<750	55	2	1	-	-
≥750	53	7	4.03	[0.80;20.38]	0.092
**Serum albumin (g/L)**					
<35	32	3	1	-	-
≥35	117	9	0.81	[0.20;3.17]	0.757
**WBC (G/L)**					
<15	140	9	1	-	-
≥15	54	6	1.82	[0.62-5.38]	0.279
**LDH (U/L)**					
Normal	47	2	1	-	-
Elevated [a]	121	10	2.03	[0.43-9.62]	0.374
**Subtype of sAML**					
MDS-related	67	10	1	-	-
MPN-related	32	2	0.38	[0.08;1.85]	0.230
Therapy-related	82	2	0.14	[0.03;0.68]	0.014
**Cytogenetics**					
Intermediate	123	8	1	-	-
Unfavorable /non monosomal	46	5	1.75	[0.54;5.66]	0.348
Monosomal karyotype	25	2	1.25	[0.25;6.27]	0.786
**Treatment arm**					
Azacitidine	104	6	1	-	-
Intensive chemotherapy	92	10	1.99	[0.69;5.71]	0.200

sAML: secondary AML; MDS: Myelodysplastic syndrome, MPN: Myeloproliferative neoplasm, WBC: white blood cell count, OR: Odds ratio, CI: confidence interval, [a]. above the benchmark

Supplementary Table 3: Univariate analysis of factors associated with response to treatment

	N	N Events	OR	95% CI	p
**Age as continuous variable**					
Interval of 5 years	199	79	0.88	[0.84 ;0.92]	<0.001
**Sex**					
Male	117	46	1	-	-
Female	82	33	1.04	[0.58;1.85]	0.895
**Performance status**					
0-1	123	49	1	-	-
2-3	49	22	1.23	[0.63;2.40]	0.543
**Ferritinemia (µg/L)**					
<750	55	26	1	-	-
≥750	53	24	0.92	[0.43;1.97]	0.836
**Serum albumin (g/L)**					
<35	32	16	1	-	-
≥35	118	48	0.69	[0.31;1.50]	0.346
**WBC (G/L)**					
<15	143	46	1	-	-
≥15	54	33	3.31	[1.73;6.35]	<0.001
**LDH (U/L)**					
Normal	48	21	1	-	-
Elevated [a]	121	51	0.94	[0.48;1.84]	0.849
**Subtype of sAML**					
MDS-related	69	19	1	-	-
MPN-related	32	10	1.20	[0.48;2.99]	0.701
Therapy-related	83	45	3.12	[1.58;6.17]	0.001
**Cytogenetics**					
Intermediate	126	57	1	-	-
Unfavorable /non monosomal	46	16	0.65	[0.32;1.30]	0.221
Monosomal karyotype	25	5	0.30	[0.11;0.86]	0.024
**Treatment arm**					
Azacitidine	107	21	1	-	-
Intensive chemotherapy	92	58	6.99	[3.69;13.22]	<0.001

sAML: secondary AML; MDS: Myelodysplastic syndrome, MPN: Myeloproliferative neoplasm, WBC: white blood cell count, OR: Odds ratio, CI: confidence interval, [a]. above the benchmark

Supplementary Table 4: Univariate analysis of factors associated with overall survival

	N	N Events	HR	95% CI	p
**Age (years)**					
<70	85	63	1	-	-
≥70	114	102	1.14	[0.83;1.56]	0.415
**Sex**					
Male	117	100	1	-	-
Female	82	65	0.90	[0.66;1.23]	0.503
**Performance status**					
0-1	123	96	1	-	-
2-3	49	43	1.46	[1.01;2.10]	0.043
**Ferritinemia (µg/L)**					
<750	55	39	1	-	-
≥750	53	45	1.74	[1.13;2;68]	0.012
**Serum albumin (g/L)**					
<35	32	25	1	-	-
≥35	118	98	1.00	[0.64;1;55]	0.995
**WBC (G/L)**					
<15	143	120	1	-	-
≥15	54	43	1.07	[0.75;1;52]	0.713
**LDH (U/L)**					
Normal	48	37	1	-	-
Elevated [a]	121	101	1.66	[1.14;2;43]	0.009
**Subtype of sAML**					
MDS-related	69	54	1	-	-
MPN-related	32	29	1.59	[1.01;2;52]	0.046
Therapy-related	83	69	1.11	[0.77;1.58]	0.581
**Cytogenetics**					
Intermediate	126	98	1	-	-
Unfavorable /non monosomal	46	43	2.52	[1.72;3.69]	<0.001
Monosomal karyotype	25	22	3.22	[1.98;5.25]	<0.001
**Allogeneic SCT**					
No	171	143	1	-	-
Yes	9	4	0.38	[0.14;1.02]	0.054
**Treatment arm**					
Azacitidine	107	94	1	-	-
Intensive chemotherapy	92	71	0.98	[0.72;1.34]	0.901

sAML: secondary AML, SCT: stem cell transplant, MDS: Myelodysplastic syndrome, MPN: Myeloproliferative neoplasm, WBC: white blood cell count, HR: hazard ratio, CI: confidence interval, SCT: stem cell-transplantation, [a]. above the benchmark
